# Construction of computer model for enterprise green innovation by PSO-BPNN algorithm and its impact on economic performance

**DOI:** 10.1371/journal.pone.0262963

**Published:** 2022-01-28

**Authors:** Xiaomei Zhang, Zhuosi Tang

**Affiliations:** School of Finance, Southwestern University of Finance and Economics, Chengdu, China; Torrens University Australia, AUSTRALIA

## Abstract

The present work aims to analyze the elements that affect corporate green technology innovation and investigate a method suitable for predicting and evaluating corporate performance. First, the elements of green technology innovation and their relationships are analyzed and explained. Then, the Complex Adaptive System (CAS) theory is introduced. On this basis, a computer model for the driving mechanism system of corporate green technology innovation is constructed on the Recursive Porus Agent Simulation (Repast) platform. Finally, the Backpropagation Neural Network (BPNN) model is optimized by Particle Swarm Optimization (PSO), constituting the PSO-BPNN algorithm to evaluate corporate performance. The results of network training and simulation demonstrate that compared with traditional BPNN, PSO-BPNN achieve a faster convergence speed and fewer errors. Besides, the actual output value has a tiny difference from the expected value, showing the application potential of this algorithm in corporate performance prediction. Moreover, the driving factors of green technology innovation greatly affect the profitability and performance of enterprises. Given insufficient corporate profit margin, continuous technological innovation activities can ensure the normal operation of enterprises. A smaller corporate tax rate can shorten the time for the system to reach equilibrium. When the corporate tax rate is above 0.2, the system takes longer to reach equilibrium. In addition, the public opinion coefficient directly affects the time needed for the system to attain equilibrium. When the public opinion coefficient is within 50,00 ~ 6,000 interval, the time that the system takes to reach equilibrium changes significantly. Furthermore, corporate internal and external driving factors have a direct effect on corporate green technology innovation and performance. The research findings indicate that the PSO-BPNN algorithm is of vital practical value to corporate performance evaluation.

## Introduction

The accelerated development of the social economy since the industrial revolution has brought huge wealth to human beings. As a result, a three-tier economic development model from resources to products and pollution emissions has been formed, which has significantly promoted the development of human society. However, with the continuous growth of population size and material demand, the society, economy and environment are constantly changing, and sustainable development has become the current global development trend [1, 2]. Green ideas and concepts are gradually introduced into production and technology innovation, and transformed into practice [3]. Green technology is a material guarantee for the circular economy, and green technology innovation is a useful measure to promote sustainable development [4, 5]. The concept of green technology innovation is in line with the requirements of current environmental development, and it is conducive to the internalization of environmental cost of enterprises. With the emergence and popularity of green products, consumption, and marketing, the concept of sustainable development has become increasingly important and attracted attention from the massive [6, 7]. Green technology innovation can trigger off the transformation and development of economic theories, management methods, and factor allocation, thereby promoting economic and social development and progress. Similarly, research on the driving mechanism of corporate green technology innovation is of great significance to promote corporate green technology innovation. The concept of Backpropagation Neural Network (BPNN) was first proposed by Rumelhart and McCelland. Due to its excellent performance, BPNN has developed into a widely used neural network. In addition to memory and learning capabilities, BPNN can also store the mapping relationship between input and output patterns [8, 9]. Hence, BPNN with applicability to the prediction field is adopted for the corporate performance evaluation that has non-linear characteristics. The present work tries to reveal the widespread contradictions and problems of Chinese industrial enterprises in green technology innovation, and conduct systematic and scientific analysis and evaluation on the importance of enhancing the technological innovation of green technology innovation. It aims to put forward countermeasures and suggestions for promoting the efficiency of green technology innovation in Chinese industrial enterprises, and provide basis for the decision-making of enterprises and policy-making of relevant departments.

Many scholars have conducted research regarding green technology innovation. For example, Abbas and Sagsan (2019) discussed the role of knowledge management in green innovation and sustainable enterprise development. They analyzed the data collected from lower, middle and upper-level managers of small, medium and large-sized manufacturing and services firms located in Pakistan through structural equation modelling to investigate how knowledge management processes. They found that knowledge management greatly affected green innovation and sustainable development, and knowledge management played an important role in manufacturing and service industries of different scales [10]. Zhang (2020) reported that technological innovation was the key to solving the contradiction between environmental pollution and economic growth, so they re-examined the relationship between environmental regulations and green technology innovation. The authors discussed the characteristics and influencing factors of government and corporate behavior by constructing an evolutionary game model. They concluded that people believed that environmental laws and regulations would affect the enterprise’s green technology decisions, and the technical level might affect policy adjustments related to environmental laws and regulations [11]. Pali and Prester (2020) evaluated whether manufacturing enterprises could improve their performance through green innovation. They adopted a three-step Ordinary Least Square for regression analysis. They proved that advanced manufacturing enterprises had contributed to corporate performance and green innovation [12]. Regarding the application of BPNN in prediction, Zhang (2019) introduced a production prediction model based on BPNN optimized by Particle Swarm Optimization (PSO) to cope with the shortcomings of predicting aquaculture production and export scale. The results of their experiments showed that the improved BPNN algorithm had smaller root mean square error, higher learning efficiency, and better prediction results than the traditional BPNN algorithm [13]. Xu and He (2018) proposed a BPNN model based on improved genetic algorithm to predict carbon dioxide content. Finally, the researchers verified the effectiveness of the proposed BPNN model through simulation experiments [14]. Xu et al. (2019) pointed out that cross domain collaborative filtering was not only a method to transfer evaluation knowledge across domains, but also a new method to effectively alleviate the sparsity of recommendation systems. In addition, most recommendation systems only used the information from the auxiliary domain from the user or project side. To overcome these shortcomings, the author proposed a cross-domain cooperative filtering algorithm which utilized the latent factor space of the auxiliary domain to expand the characteristics of users and items. First, they presented the recommendation problem as the classification problem of the target domain, with the location of users and goods as the feature vector and the rating of users and goods as the label through this algorithm. Then, they used funk singular value decomposition to extract extra user and item features from user- and item-side auxiliary domains, respectively, to expand the two-dimensional position feature vector. Finally, they trained a classifier using the C4.5 decision tree algorithm to predict missing ratings. Through experiments, they proved that this algorithm could make full use of user- and item-side information [15]. Olagunju (2019) reported that the current and emerging networks had the responsibility to promote new cooperative research and commercial interconnection between academia and industry. The authors also believed that multi-layer networks needed to describe the relationship between nodes from different network nodes, so that different organizations could cooperate in research activities such as data mining. In addition, they proposed an algorithm to find interference between communication nodes in multi-layer networks [16]. Ye and Yan (2018) [17] designed a simple and effective least squares double bounded support vector machine based on the classical double bounded support vector machine. This algorithm reconstructed the inequality constraints of the double bounded support vector machine problem by using the equality constraints, which reduced the difficulty of solving a pair of least squares problems. The authors derived two optimal nonparallel planes by solving only a pair of linear equations. They proved that compared with doubly bounded support vector machines, the least squares doubly bounded support vector machines had lower time complexity and could deal with large data sets more efficiently. In short, there is already lots of research on using green technology innovation and BPNN for prediction. However, studies that explore the driving mechanism of enterprises’ green technological innovation are rarely reported. Most scholars have conducted comprehensive evaluation research on the result performance of the research object based on the systematic analysis of green technology innovation. The comprehensive evaluation can reflect the idea of comprehensively grasping the current situation of green innovation performance in the green innovation system. However, there are problems of excessively rough evaluation index systems, mediocre indicators on the whole, and the unclear key factors due to the too broad dimension of consideration. Moreover, it is difficult to construct a comprehensive evaluation index system, or to fully follow the scientific and reasonable selection principle, and the accuracy of the evaluation is questioned.

Therefore, a green technology innovation power mechanism model is proposed based on the Complex Adaptive System (CAS) theory to explore the composition and driving factors of corporate green innovation and seek a method suitable for enterprise performance prediction. Moreover, a computer model of the power mechanism of the enterprise green technology innovation system is constructed using the Recursive Porus Agent Simulation (Repast) platform. In addition, the BPNN model optimized by PSO, denoted as PSO-BPNN in the present work, is employed for enterprise performance prediction, which can provide a reference for the sustainable development and innovation activities of the enterprise.

## Method

### Green technology innovation and driving factor recognition

**Fig 1:**
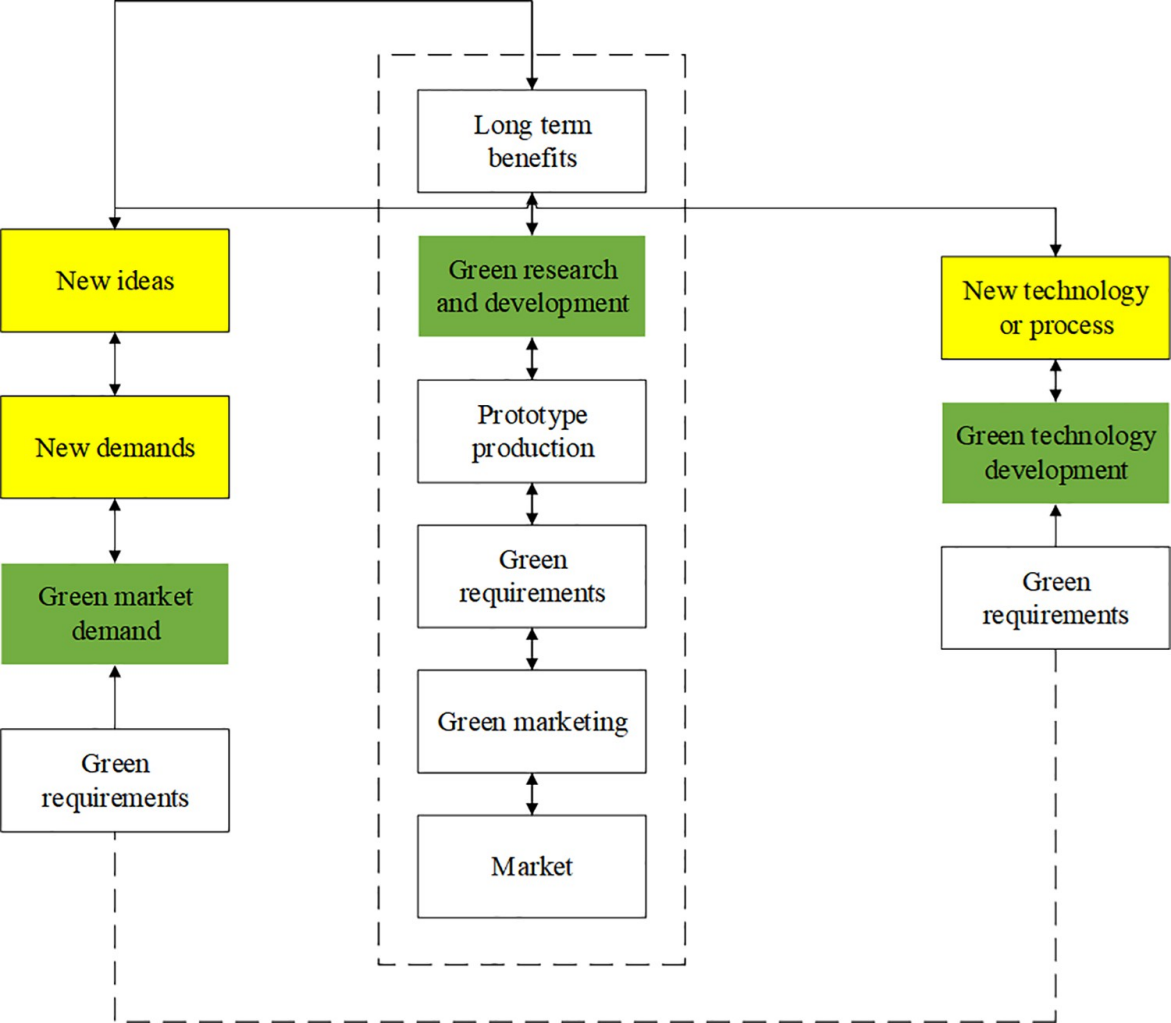
The overall implementation of green technology innovation.

The concept of green technology was first proposed in the 1960s. This terminology reveals the relationship between humans and nature while considering the balance between environmental pollution and natural ecology to facilitate the coordinated development of environmental resources. From an overall evolution perspective, green technology can be divided into end treatment, cleaning, and green products, which reflects the different depths of the technology. In terms of characteristics, green technology actually represents those technologies that can reduce pollution, save energy, and reduce consumption. Green technology innovation is developed based on the concept of green technology. Unlike the traditional form of technological innovation, green technology innovation is a brand-new reform. Green technology innovation can enhance economic benefits and environmental value and improve environment by combining environmental protection with green technology. Green technology innovation is actually a form of integrated innovation based on the whole product life cycle, which pursues the maximization of economic, ecological and social benefits. The overall implementation of green technology innovation is displayed in Fig 1.

**Fig 2:**
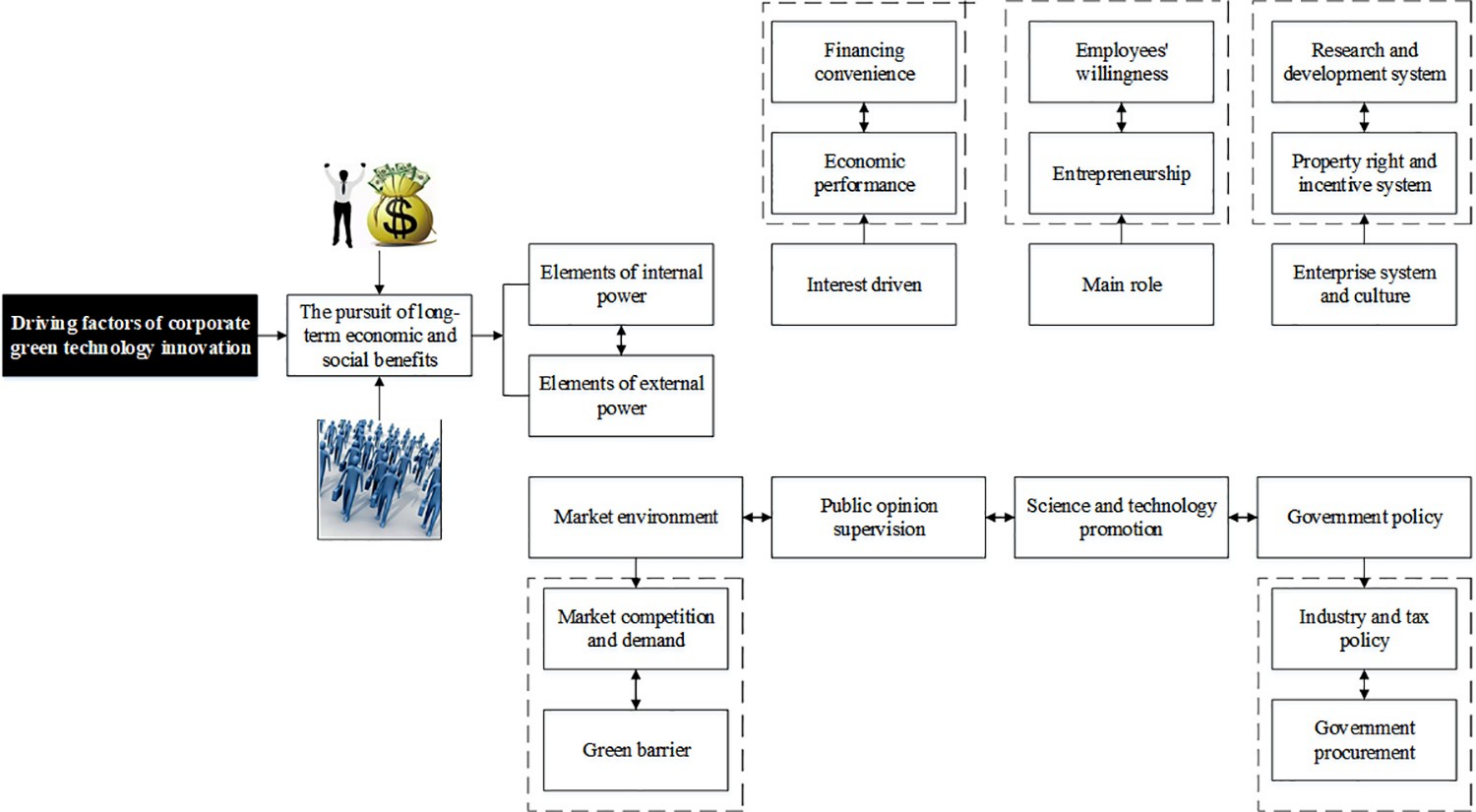
Relationship and recognition of corporate green technology innovation’ driving factors.

Due to the unique form of green technology innovation, it is difficult to use one element to clearly illustrate the driving force of an enterprise’s green technology innovation. For enterprises, the driving force of green technology innovation is fundamentally their pursuit of maximum economic and social benefits. Specifically, the driving force comes from inside and outside the enterprise [18]. The internal driving factors of green technology innovation of an enterprise include continuous innovation consciousness, spirit, profit-driving, culture, and system. The external driving factors of an enterprise’s green technology innovation involve technology promotion, market mechanism, economic culture, and political system. In terms of internal driving factors, the economic benefits pursued by enterprises are the foundation of technology innovation activities. Whether the expected benefits of technology innovation driven by interests will be transformed into innovation power is inseparable from the enterprise’s goals. Enterprises are the principal leaders in not only the market economy but also technology innovation. Besides, an enterprise is a group of employees and entrepreneurs, and the overall interests of the enterprise is the combination of the interests of all parties. The innovation culture of the company serves enterprise innovation, including innovative spirit, atmosphere, and tool system. The enterprise system has a close relationship with innovation revenue. The development of a driving mechanism system of corporate green technology innovation is essential to promote enterprise innovation activities. The above factors are all internal influencing factors of green technology innovation activities of enterprises. The market environment and market demand determine the foundation and driving source of innovation activities as external factors. Technology improvement is a powerful promoter of technology innovation activities that usually begin with research and development. Government policies and social supervision are also critical driving factors to develop innovative activities. Fig 2 illustrates the relationship and recognition of the driving factors of enterprise green technology innovation.

### Construction of the driving mechanism model of green technology innovation

The above analyses suggest that driving factors that affect corporate green technology innovation are numerous, and their relationships are rather complicated. Hence, the CAS theory [19] is introduced to discuss the driving mechanism of corporate green technology innovation. Although differences exist between different complex systems, they all consider the coordinated development of the system. Models included in the CAS theory are the stimulus-response model and the echo model. The former represents the basic behavior of the subject in various systems, which comprises detectors, effectors, and IF/THEN rules. The detector characterizes the subject’s ability to obtain information; the effector represents the subject’s reaction to the environment; the IF/THEN rule reflects the subject’s ability to process information. The echo model consists of offense identification, defense identification, and resource library. The CAS theory can adequately describe the evolution process of things from simple to complex using the above two models; meanwhile, it can clearly characterize the evolution mechanism of complex systems.

**Fig 3:**
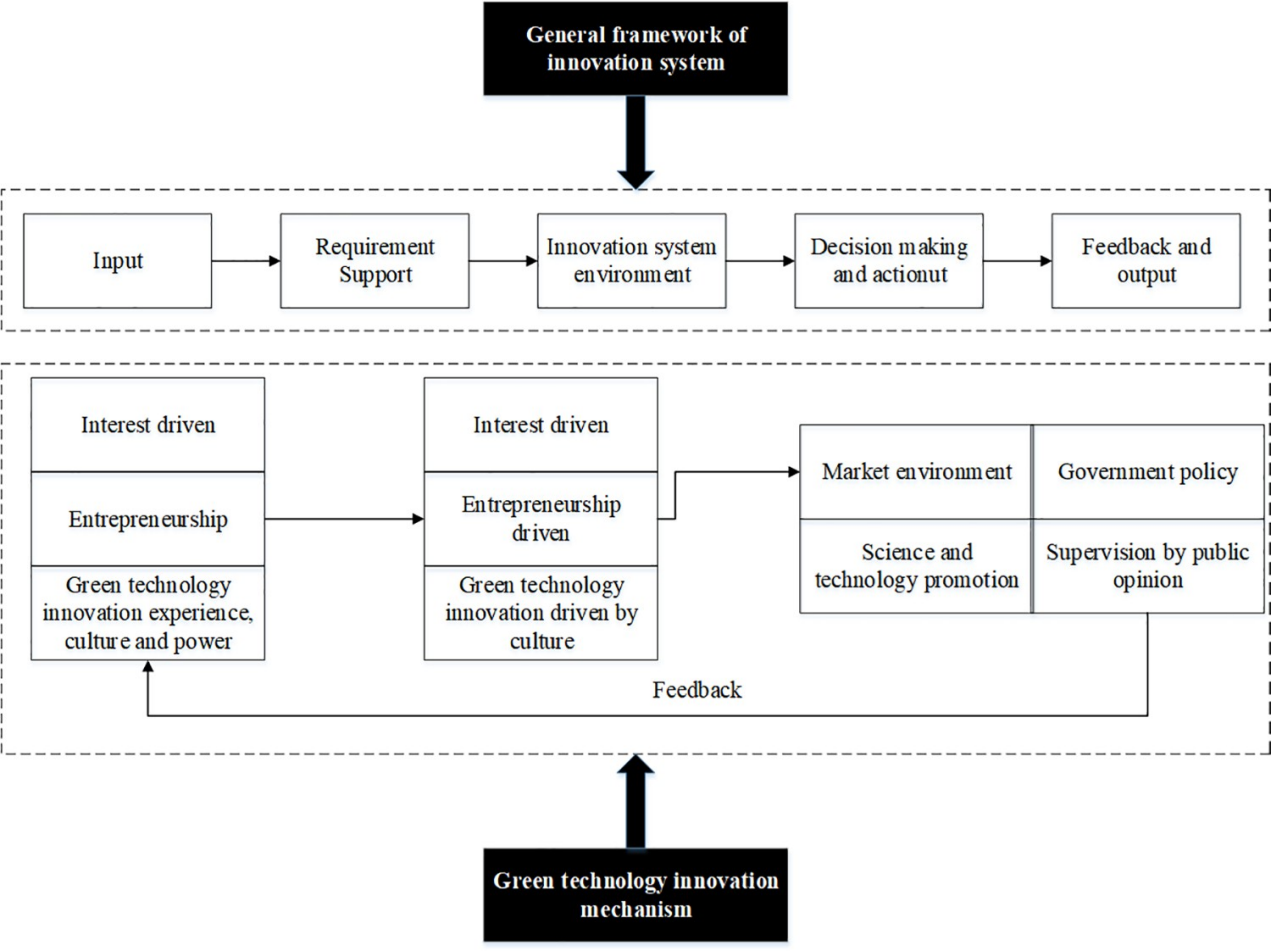
The general framework of the innovation system and the driving mechanism system of green technology innovation.

A system is a synthesis of numerous elements, and diversity and correlation are characteristics of systems. Diversity reflects the differences of various elements in the system, while correlation reveals the interdependence and connection of the various components in the system. Hence, simplifying the study of the complex systems in an interrelated way is feasible, which can be referred to as a system mechanism. In this regard, the green technology innovation mechanism is chosen to simplify the research on the green technology innovation system. The so-called green technology innovation mechanism refers to the functional structure and interaction mode of various components within the system. The corporate innovation experience, culture, ability, and other factors all affect the behavior of the corresponding technology innovation community. It is believed that the technological innovation culture, innovation capabilities, and innovation experience shared by the enterprise have a dominating effect on the corporate green technology innovation mechanism. Besides, this effect is a solidified state of the green technology innovation system and mechanism, affecting the entire innovation system’s structural behavior. The structural behaviors will, in turn, affect the perception, evaluation, and orientation of green technology innovation. Fig 3 displays the general framework of the innovation system and the driving mechanism model of green technology innovation.

### Corporate performance prediction model based on PSO-BPNN

**Fig 4:**
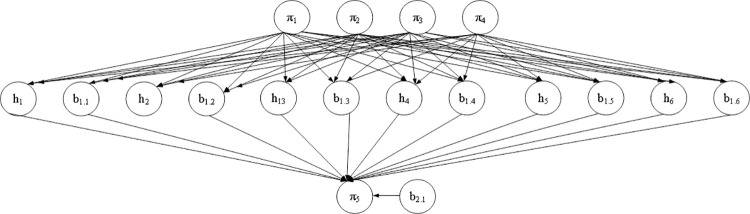
Structure of the BPNN model.

BPNN is an intelligent network characterized by adaptive learning and memory, which includes the input layer, the hidden layer, and the output layer. Data is input into the input layer and trained in the hidden layer. The output layer outputs prediction or evaluation results. At the same time, the network can minimize the output error by comparing the output with the expected value through backpropagation. If there is any inconsistency between the value obtained by the model output and the expected value, BPNN feedback an error along the original path to modify the corresponding weight value. BPNN carries out above processing cycles alternately to obtain the ultimate value with the minimum error, which completes the learning process. Although BPNN has been extensively used, it still has some shortcomings summarized as the following four aspects. First, BPNN has a slower convergence speed and longer training time. Second, BPNN can converge the weight value to a set value through error correction; however, it is challenging to ensure that the obtained error value is the global minimum simultaneously, because multiple local minimum values may be obtained using the gradient descent method. Third, BPNN has strong redundancy. Fourth, BPNN is unstable in the learning and memory process, lacking memory function for weights and thresholds. Fig 4 shows the structure of the BPNN model.

PSO is inspired by the migration and foraging process of birds. Like the genetic algorithm, PSO is an intelligent evolutionary algorithm based on a random value that finds the optimal solution through continuous iterations [20, 21]. According to the fitness value, PSO can evaluate the solution in a simpler way than the genetic algorithm. Therefore, the PSO algorithm is suitable for dealing with high-dimensional optimization problems with multiple extreme values and low accuracy requirements. The implementation of the PSO algorithm does not require adjustments of complicated parameters. PSO plays a significant role in neural network optimization and fuzzy control. Here, the PSO algorithm is applied to improve and optimize BPNN.

The iterations of the corresponding speed *v* and position *p* of the PSO algorithm are expressed as:

$$v_{id}{}^{\left(t+1\right)}=wv_{id}{}^{\left(t\right)}+c_{1}r_{1}(p_{id}-x_{id}{}^{t})+c_{2}r_{2}(p_{gd}-x_{id}{}^{t})$$ (1)

$$p^{\left(t+1\right)}{}_{id}=p^{\left(t\right)}{}_{id}+v_{id}{}^{\left(t+1\right)}$$ (2)

where *w* denotes the inertia weight, *c*_*1*_ and *c*_*2*_ refer to the learning factors, and *r*_*1*_ and *r*_*2*_ stand for the random numbers in the interval (0, 1). The PSO algorithm can adjust the global and local optimization capabilities by introducing the inertial weight, thereby avoiding falling into the local optimum. The inertia weight *w* is calculated according to Eq (3).


$$w=w_{\max}-((w_{\max}-w_{\min})/t_{\max})*t_{i}$$ (3)

In Eq (3), *w*_*max*_ represents the maximum inertia weight set at the beginning, *w*_*min*_ denotes the corresponding minimum inertia weight, *t*_*max*_ refers to the maximum iteration times of the population, and *t*_*i*_ stands for the cycle iteration.

Assuming that the weight of the BPNN is the position of the particle in the PSO algorithm, then, two consecutive changes in the weight value can be regarded as the changes in the position of the particle in BPNN’s training process. On this basis, the corresponding weight changes of BPNN can be written as:

$$\Delta w_{kj}=wv_{id}+c_{1}r_{1}(w_{kj}(b)-w_{kj})+c_{2}r_{2}(w_{kj}(g)-w_{kj})$$ (4)

$$\Delta w_{ji}=wv_{id}+c_{1}^{'}r_{1}^{'}(w_{ji}(b)-w_{ji})+c_{2}^{'}r_{2}^{'}(w_{ji}(g)-w_{ji})$$ (5)

where *w*_*kj*_(*b*) and *w*_*ji*_(*b*) stand for the BPNN weights under the optimal conditions of the individual, and *w*_*kj*_(*g*) and *w*_*ji*_(*g*) refer to the optimal conditions of the swarm weights of BPNN.

The excellent performance of BPNN in adaptability, fault tolerance, and robustness has significantly expanded its application range, and BPNN is feasible to predict and evaluate corporate performance. When constructing the evaluation model, the key points include establishing an evaluation indicator system, selecting sample data, and training the network to learn and seek the relationship between the input value and the output value, thereby obtaining the optimal solution. During the indicator system establishment, the data after normalization is input into BPNN, and then transmitted to the output layer after the processing of the hidden layers through forward propagated. Finally, the output layer obtains the processing result. Furthermore, BPNN compares the output value with the expected value. if any error exists, the training and learning will not be terminated until the error is smaller than a determined threshold through adjustment and correction. BPNN can evaluate corporate performance using the sample data obtained from training. In the training of BPNN, multiple enterprises from eight Chinese cities are selected as training samples. Among them, the hidden layer of BPNN can be determined according to Eq (6).


$$n_{l}=\sqrt{n+m}+a$$ (6)

In Eq (6), *n*_*l*_ represents the number of hidden layer nodes, *n* denotes the number of input layer nodes, *m* stands for the number of output layer nodes, and *a* refers to a constant in the range of 1–10.

The neural network is trained in the MATLAB toolbox using TRAINLM as the training function, LEARNGDM as the adaptive function, MSE as the performance function, and TANSIG as the transfer function.

### Simulation model design

Repast is a subject-based simulation platform developed using Java. This framework provides multiple class libraries creating, collecting, and processing the simulation data [22]. Enterprises are taken as the subject on the Repast platform where the innovation space is defined, so that the subject can be projected into the innovation space. The Repast platform serves as the foundation to simulate corporate operation in a regional environment for the corporate green technology innovation system. The area in the simulation system includes a specific number of enterprises, decision-making departments, social environment, and markets. Taking technology and capital as independent variables, Repast establishes the production function in the computer model of the driving mechanism system of corporate green technology innovation, as presented in Eq (7).


Q=F(C,T) (7)

In Eq (7), *Q* represents the output quantity, *C* stands for the input capital of the production process, and *T* refers to the technical level.

Besides, due to the internal and external factors that affect corporate green technology innovation, the qualitative function of the driving mechanism model is expressed as Eq (8).


P=f(pr,sp,cu,i,ca,m,sc,po,su) (8)

In Eq (8), *pr* indicates profit-driven, *sp* indicates entrepreneurial spirit, *cu* stands for corporate innovation culture, *i* stands for internal incentives, and *ca* refers to technological innovation capability. Besides, *m* refers to the market environment, *sc* denotes the science and technology environment, *po* denotes the policy environment, and *su* represents the public opinion supervision. Eq (8) is a qualitative functional equation, among which an interest driver is selected as a variable for subsequent analysis.

**Fig 5:**
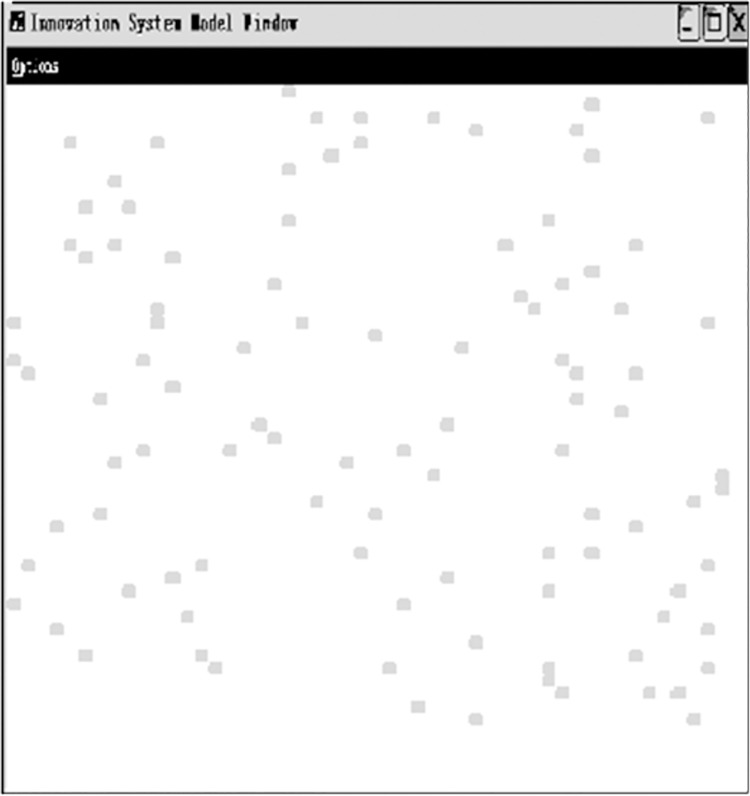
Distribution of enterprises in the computer model.

Based on the above key parameters, the elements of enterprise performance can be analyzed by changing the technical environmental conditions and applying the PSO-BPNN model. The selected enterprises are sequentially distributed in eight cities represented by A-H. The six cities selected for the neural network test are denoted by I-N in turn. Fig 5 revels a computer model of enterprise distribution.

The computer model can provide an overall perception of the distribution of enterprises. The relatively concentrated distribution of enterprises shows that the research area has particular representativeness, and the data collected are comprehensive and true.

### Data set and parameter configuration

The sample of this survey is selected from the electronic component manufacturing industry of the electronic industry in the industry classification of the China Securities Regulatory Commission. The sample data is the data given by the listed companies in the electronic component manufacturing industry in 2020. Excluding the companies without basic financial data or incomplete data, 15 of the remaining 23 companies are selected as the sample. In addition, 10 indicators are selected, namely return on net assets, earnings per share, cost-cost margins, accounts receivable turnover, total asset turnover, inventory turnover, current ratio, cash-current liabilities ratio, total asset turnover, and operating profit growth rate. The actual values of each indicator are normalized. Since the input data of BPNN must be digitized, and the value range of input variables has special requirements, the data collection results of all evaluation indicators must be preprocessed. Here, all indicator values are digitized and normalized respectively according to digital statistics and expert experience. Eq (9) describes the normalization method.


$$V=\frac{x‐x_{min}}{x_{max}‐x_{min}}$$ (9)

In Eq (9), *V* represents the result value of normalization, while *x*_*min*_ and *x*_*max*_ denote the minimum and maximum values of an indicator, respectively.

## Results and discussion

### Training results of the neural network

**Fig 6:**
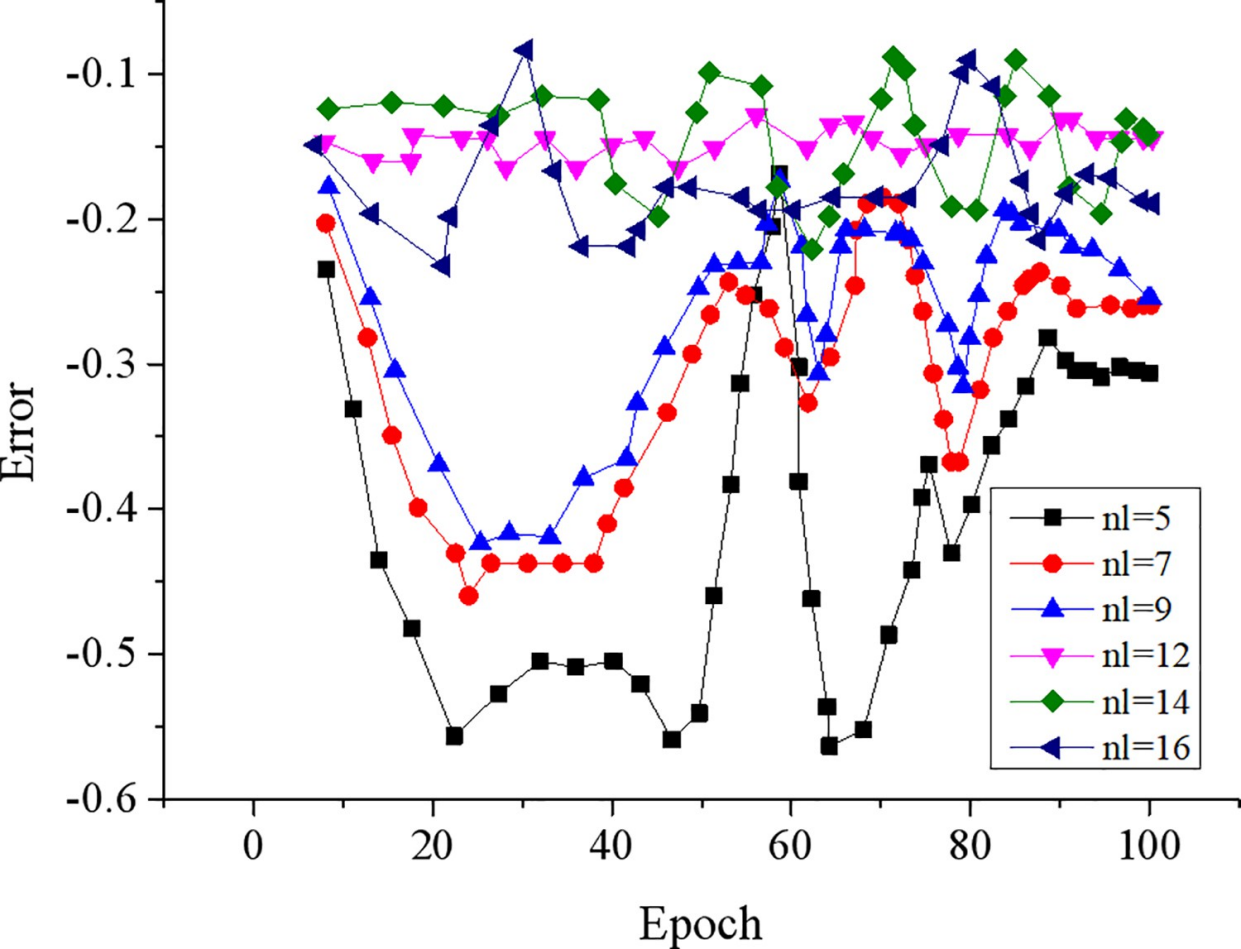
Error curves of BPNNs under different number of hidden layer nodes.

The errors of different BPNN models when the number of hidden layer nodes are 5, 7, 9, 12, 14, and 16 are compared, as displayed in Fig 6.

According to the changes and distribution of errors, BPNN with 12 hidden layer nodes achieves the smallest error. Hence, the number of hidden layer nodes of BPNN is determined to be 12.

**Fig 7:**
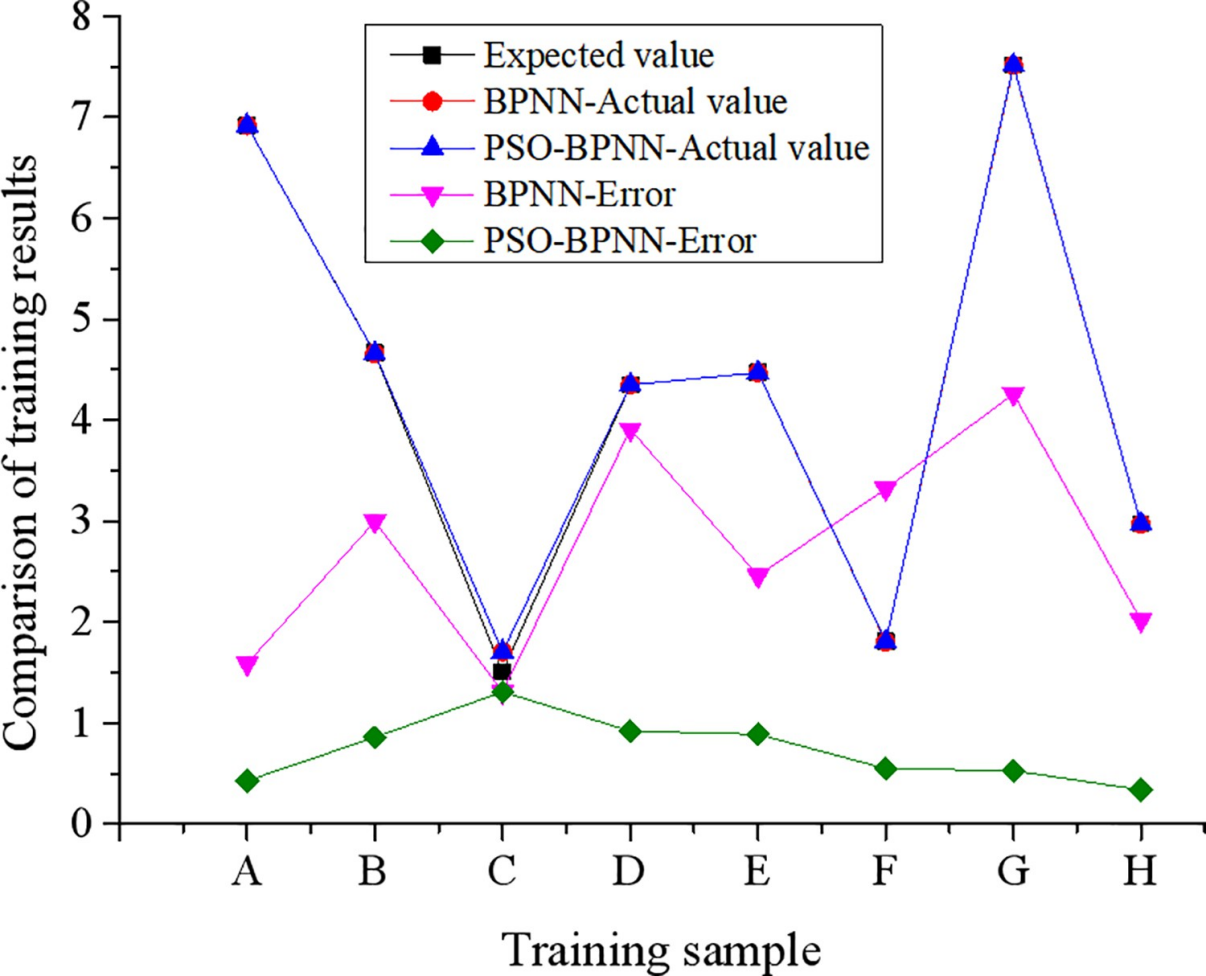
Training results of BPNN before and after optimization.

**Fig 8:**
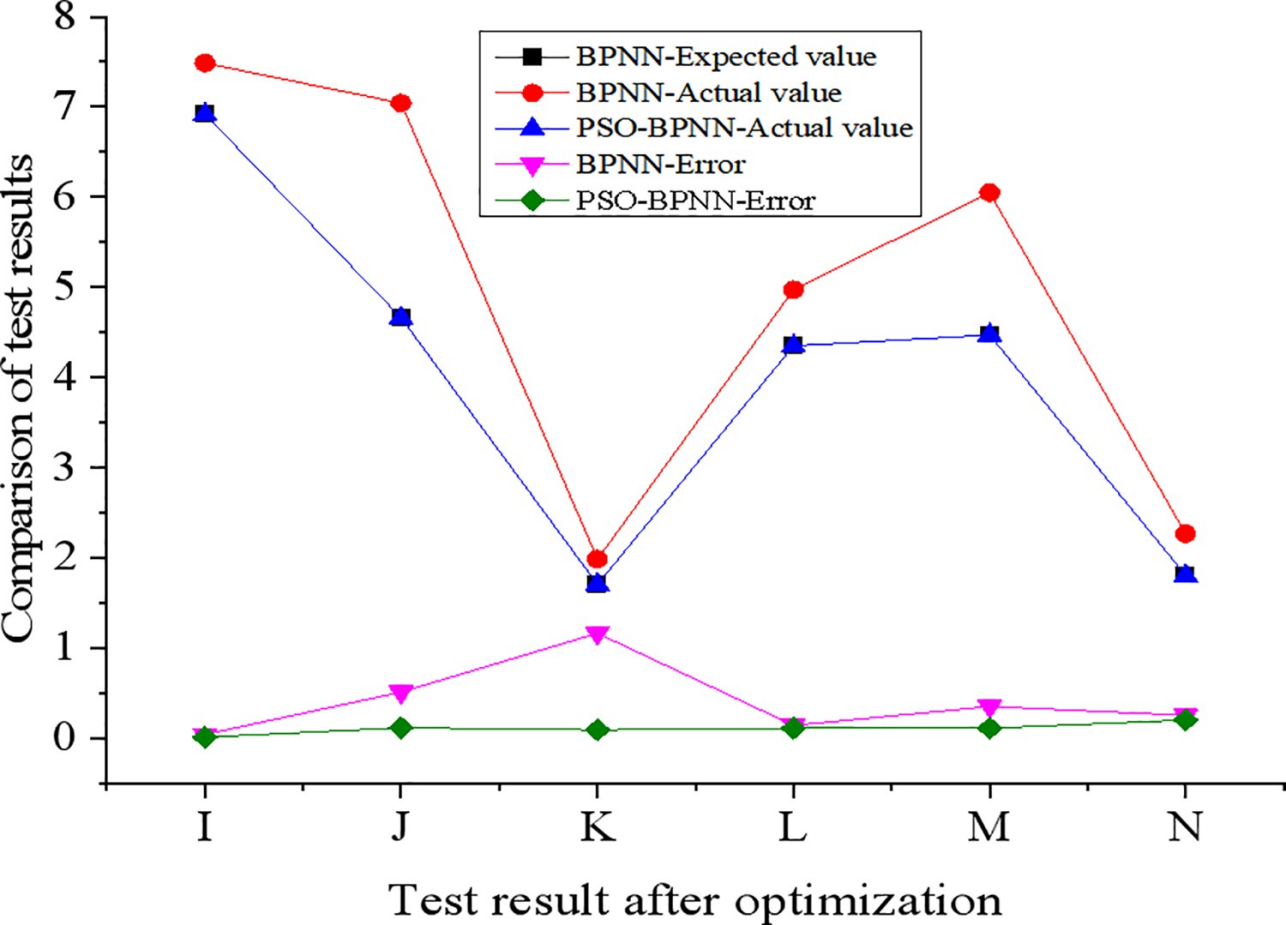
Testing results of BPNN before and after optimization.

The training results and test results of BPNN before and after optimizing by PSO are presented in Figs 7 and 8.

Apparently, BPNN provides higher accuracy during the training process; however, the error becomes larger, and the accuracy decreases during the test process. The reason maybe the selection of fewer test samples. Nevertheless, BPNN has application potential in evaluating and predicting corporate performance. The results in Figs 7 and 8 demonstrate that the error value of the PSO-BPNN is significantly reduced than BPNN without optimization. Through comparison, the error value of the PSO-BPNN model is significantly reduced than BPNN. Compared with traditional BPNN, PSO-BPNN is more suitable for predicting enterprise performance. The reason is that traditional BPNN is easy to fall into local optimum [23], but PSO can exactly avoid this problem. Moreover, the PSO-BPNN model has a faster convergence speed and smaller error between the actual value and the expected value.

### Simulation results of internal driving factors of enterprises

**Fig 9:**
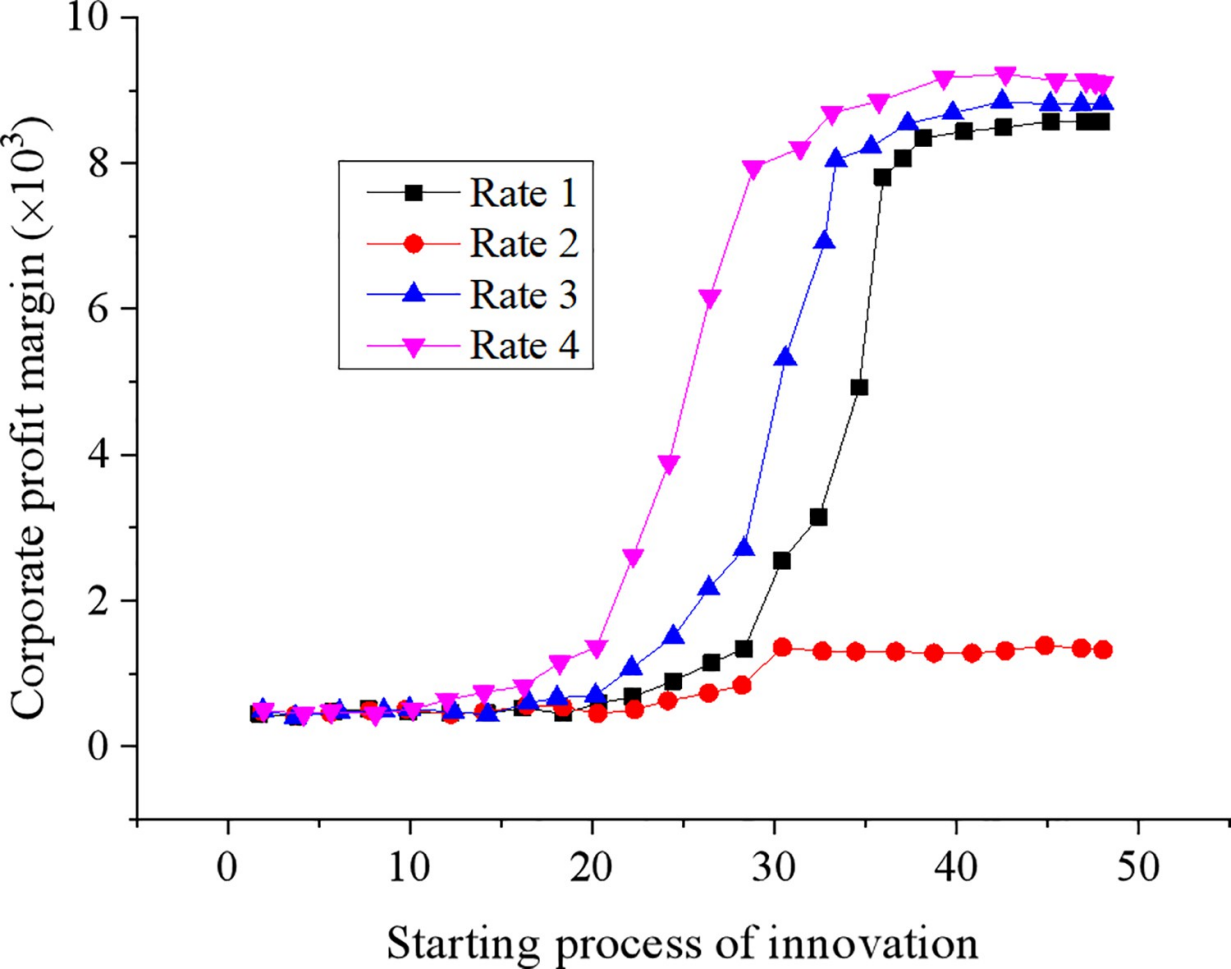
The relationship between the starting process of corporate innovation and corporate profit margin.

The relationship between the starting process and the performance of corporate innovation is obtained based on the corporate profit margin and the driving mechanism computer model of the corporate green technology innovation system proposed above. The results are shown in Fig 9.

Data changes in Fig 9 indicate that with the increase in the market economy and competition intensity driven by innovation, the corporate capital return rate shows a downward trend. According to the size of the capital profit margin that different enterprises can accept, corporate innovation activities can be started. When the effect of market competition on corporate performance has not yet reached the threshold level, enterprises with lower competitive pressure will not take the initiative to start green innovation economic activities.

The corporate profit margin is linked to corporate performance.

To cope with the dilemma caused by the decrease in their profit margin, enterprises must continue technological innovation activities and promote their technical levels. The above analyses can conclude that to encourage enterprises with lower profit margin and performance to start green innovation activities, the standard of corporate innovation can be changed. The entrepreneur spirit, internal corporate incentives, and corporate culture can serve as the principal driving mechanism. Among them, the entrepreneur’s attitude towards green technology innovation is inextricably linked to the timing of corporate innovation.

### Simulation results of external driving factors of enterprises

**Fig 10:**
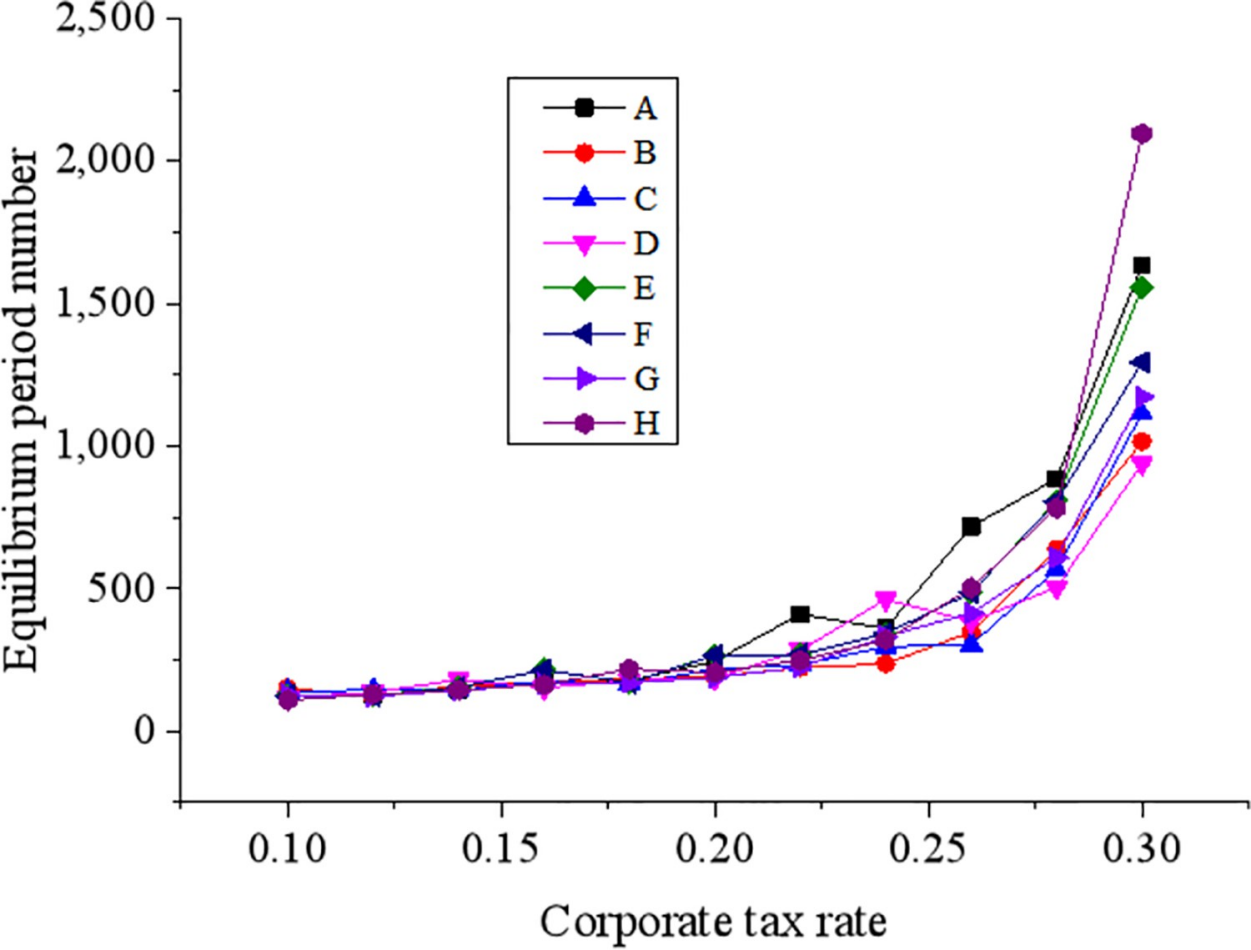
The influences of the corporate tax rate on corporate green economic innovations.

The influences of the corporate tax rate, an external driving factor of green technology innovation, on corporate green economic innovations are explained in Fig 10.

The results in Fig 10 demonstrate that the government-set tax rate for innovation products directly affects the equilibrium time of the corporate green technology innovation system. Under a lower tax rate, the system reaches equilibrium quicker. When enterprises undertake a higher tax rate, the system takes longer time to attain equilibrium. Besides, the rate change corresponding to the time required for reaching equilibrium by the system is not uniform. When the corporate tax rate is greater than 0.2, the time for the system to reach equilibrium increases significantly.

Evidently, under a lower government tax rate, corporate green technology innovation can develop faster. On the contrary, a higher government tax rate has an adverse effect on corporate green technology innovation. More severely, an extremely high tax rate can even cause an enterprise to disappear from the system. When the profit margin of a corporate product drops below the corporate minimum profit, this enterprise will start corresponding innovation activities. When the tax rate reaches a set value, green technology innovation products are more likely to be profitable. In this situation, the frequency of innovation becomes faster, and corporate pressure is also greater. According to the above results, when the tax rate that the enterprise undertakes is close to the profit margin of its products, the enterprise is more likely to start innovation activities.

**Fig 11:**
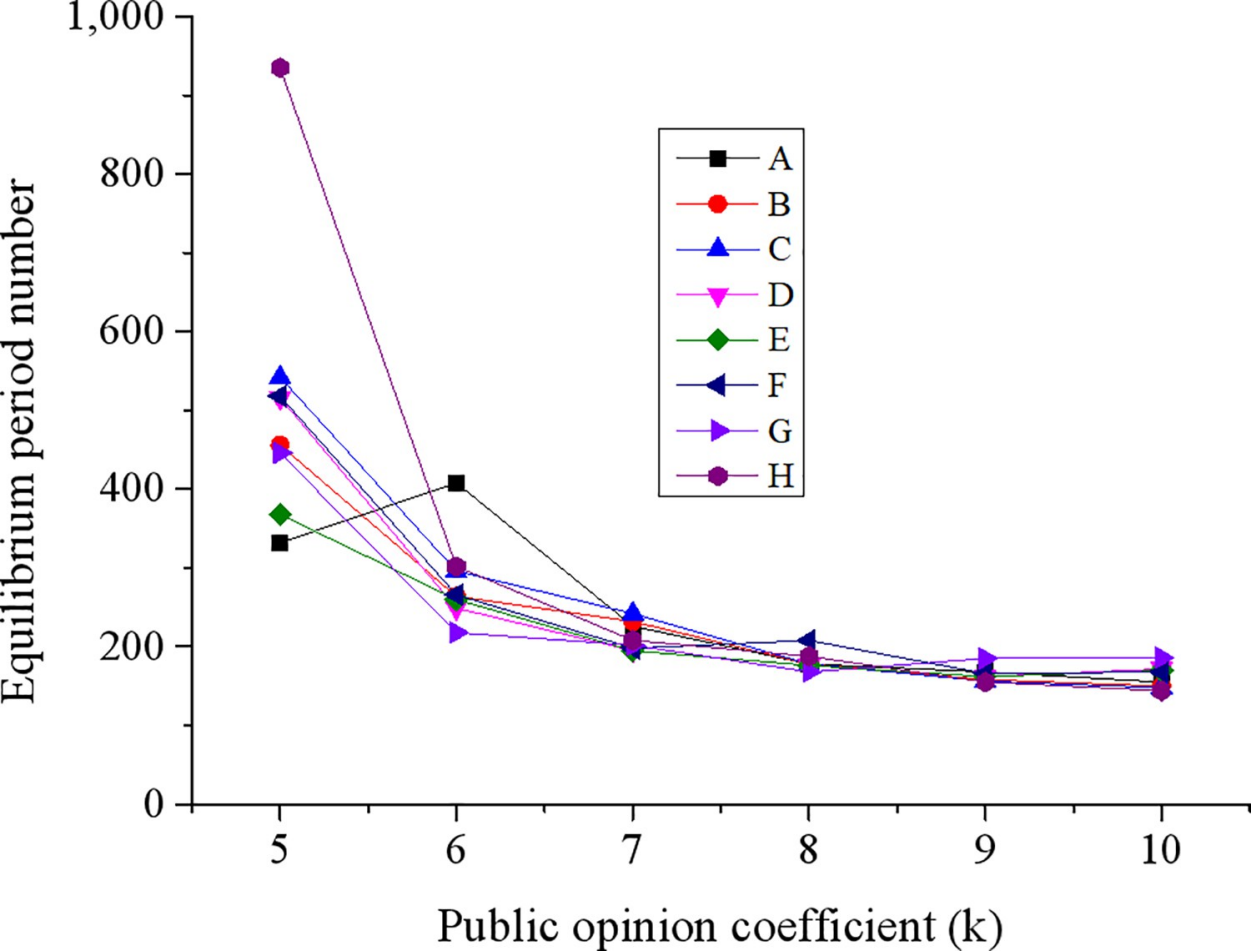
The influences of public opinion on corporate green economic innovations.

The influences of public opinion, another external driving factor of green technology innovation, on corporate green economic innovations are explained in Fig 11.

Data changes in Fig 11 suggest that as the public’s tolerance for environmental pollution decreases, the time for the system to reach equilibrium increases significantly. When the public opinion coefficient changes within the 5,000 ~ 6,000 interval, the corresponding equilibrium time rate also changes significantly.

The reason is that public opinion is a manifestation of the public’s attitude towards products. It is believed that public opinion directly affects market capacity, and the pollution brought by various products is directly related to public tolerance. When the products’ pollution reaches the highest level, the market capacity is also saturated. Consequently, those non-green products cannot finish a series of sales operations. The coefficient of public opinion can lead to changes in corporate profits and performance, and products with heavier pollution would reach market saturation rapidly. In comparison, products with less pollution are likely to make the enterprise reach a state of continuous profitability, thereby promoting corporate development and corporate innovation level.

### Comparison of algorithms

A comparative experiment is conducted to illustrate the effectiveness of the PSO-BPNN algorithm to evaluate the impact of enterprise economic performance. Convolutional Neural Network (CNN), two-dimensional CNN (2D-CNN), Long-Short Term Memory (LSTM), and Bi-directional Long Short-Term Memory (Bi-LSTM) are selected as comparative algorithms on the same computing platform. Computing platform parameters are as follows: Intel I3 2120, 2GB DDR3, AMD Radeon HD7450, windows XP. The implementation results are summarized in Table 1.

**Table 1:** Comparison of evaluation performance of different algorithms.

	PSO-BPNN	CNN	2D-CNN	LSTM	Bi-LSTM
Evaluation accuracy (%)	93.5	89.2	90.2	91.5	93.6
Time consumption (s)	13	80	70	110	170

According to Table 1, the accuracy of the PSO-BPNN algorithm on the impact evaluation of enterprise economic performance is 93.5%. This result is much higher than that of CNN algorithm, 2D-CNN algorithm, and LSTM algorithm, but slightly lower than that of Bi-LSTM algorithm. Besides, the PSO-BPNN algorithm takes 13 seconds, which is much faster than the CNN algorithm, 2D-CNN algorithm, LSTM algorithm, and Bi-LSTM algorithm. To sum up, the PSO-BPNN algorithm reported here is applicable to occasions with high requirements on time consumption and accuracy.

## Conclusion

Here, a driving mechanism model for green technology innovation is constructed based on CAS theory by elaborating the driving factor recognition of green technology innovation. Besides, a driving mechanism model for corporate green technology innovation is designed on the Repast platform. Moreover, the applicability of the PSO-BPNN model in predicting and evaluating corporate performance is analyzed. Furthermore, the training process of the neural network model reveals the applicability of PSO-BPNN to corporate performance prediction and evaluation. In particular, PSO-BPNN has a small prediction error and a fast convergence speed. The simulation results based on the internal driving factors of corporate profit margin show that enterprises with a decreasing profit margin should continue technological innovation activities. Besides, the government tax rate and public opinion can affect the development of corporate green technology innovation activities. A high tax rate increases the time that the system takes to reach the equilibrium; on the contrary, a low tax rate reduces the time to reach the equilibrium of enterprises. The public opinion coefficient also significantly changes the equilibrium time rate. The above results provide preliminary guidance for developing corporate green technology innovation activities, and a neural network model applicable to the predictive evaluation of corporate performance is realized.

However, the actual application of the PSO-BPNN algorithm in predicting corporate performance has not yet been covered. Due to the time limitation, only a few representative internal and external driving factors that affect corporate green economy innovation activities are analyzed and discussed. In future work, the practical application of PSO-BPNN will be explored, and other internal and external driving factors will be included for a more detailed analysis.

## Supporting information

S1 Data (ZIP)
